# Association of Shift, Day, Month and Year with Mortality: Observational Study of Spanish and USA Emergency Care Cohorts

**DOI:** 10.3390/medsci14010056

**Published:** 2026-01-22

**Authors:** Álvaro Astasio-Picado, José Luis Martín-Conty, Begoña Polonio-López, Cristina Rivera-Picón, Maria Eugenia Medina Chozas, Maria del Mar Palazuelos Diaz, Laura Mordillo-Mateos, Francisca Torres-Falguera, Laura Ros Gomez, Paula Alvarez Buitrago, Francisco Martín-Rodríguez, Ancor Sanz-García

**Affiliations:** 1 Intensive Care Unit, Hospital Virgen del Puerto, 10600 Plasencia, Spain; alvaro.astasio@uclm.es; 2 Faculty of Health Sciences, University of Castilla-La Mancha, Avda. Real Fábrica de Seda, s/n, 45600 Talavera de la Reina, Spain; joseluis.martinconty@uclm.es (J.L.M.-C.); begona.polonio@uclm.es (B.P.-L.); francisca.torres@uclm.es (F.T.-F.); 3 Technological Innovation Applied to Health Research Group (ITAS Group), Faculty of Health Sciences, University of Castilla-La Mancha, 45600 Talavera de la Reina, Spain; 4 Evaluación de Cuidados de Salud (ECUSAL), Instituto de Investigación Sanitaria de Castilla-La Mancha (IDISCAM), 45071 Toledo, Spain; 5 Gerencia de Urgencias Emergencias y Transporte, Servicio de Salud de Castilla-La Mancha (SESCAM), 45071 Toledo, Spain; memedina@sescam.jccm.es (M.E.M.C.);; 6 Servicio Madrileño de Salud, Hospital Universitario Puerta de Hierro, 28222 Madrid, Spain; lg1501@hotmail.com; 7 Anaesthesia and Resuscitation Service, Complejo Hospitalario de Toledo, 45007 Toledo, Spain; 8 Faculty of Medicine, University of Valladolid, 47005 Valladolid, Spain; 9 Advanced Life Support, Emergency Medical Services (SACYL), 47007 Valladolid, Spain

**Keywords:** emergency medical services, mortality, shift, month, days

## Abstract

**Background/Objectives**: Emergency medical services (EMSs) are essential for reducing mortality among critically ill patients. This study aims to evaluate the influence of temporal factors, such as time of day, day of the week, month, and year, on mortality in EMS activations, comparing health systems in the U.S. and Spain. **Methods**: This multicenter observational study, which is based on two databases (Spain’s Sacyl and the U.S.’s NEMSIS), analyzed EMS activation in high-priority adult patients (>18 years) between 2018 and 2023. Demographic variables, transport characteristics, and response times were included. Short-term mortality was the primary outcome. **Results**: A total of 54,981 EMS activations (11,713 from the Sacyl dataset and 43,268 from the NEMSIS dataset) were analyzed. Mortality was higher among older patients and males, with significant increases during shifts from 06:00 to 12:00 and from 18:00 to 24:00. Mortality also varied by year, with higher rates in 2022 and 2023 than in 2018. Notable differences were observed between the U.S. and Spain, especially in shifts and months, with higher mortality during the 12:00 to 18:00 shift and in October in the NEMSIS cohort. **Conclusions:** These findings have direct implications for emergency medical service operations, suggesting that resource allocation, staffing models, and clinical protocols should be strategically optimized based on temporal risk patterns to improve patient outcomes during identified high-risk periods.

## 1. Introduction

Emergency medical services (EMSs) play a crucial role in reducing mortality in critically ill patients, where the speed and effectiveness of prehospital care are decisive for survival. The efficiency of these systems depends on various factors, with the proper management of material and human resources being essential to ensure timely responses in emergency situations [1].

Most studies have documented the impact of saturation on mortality, revealing that EMS overload can compromise both the quality and speed of care [2,3,4]. However, this is not the only factor influencing clinical outcomes. While an increase in demand may cause delays, mortality in the out-of-hospital setting is also affected by other elements, such as shift organization, resource availability, and the structure of the healthcare system [5].

In this context, the organization of healthcare professionals’ shifts may influence patient outcomes in emergency care [6,7]. Previous studies have described temporal variations in outcomes across night shifts and weekends in emergency settings, frequently attributed to differences in workload, staffing levels, access to diagnostic resources, and system-level organization. However, findings regarding the existence and magnitude of a “weekend effect” in prehospital emergency medical services are inconsistent, and its applicability to EMS activations remains unclear. Therefore, examining temporal patterns (shift, day of week, month, and year) in prehospital mortality across different health systems is warranted [8].

Moreover, the month and year when care is provided can also influence health outcomes and, consequently, mortality. Seasonal variations, such as the increase in respiratory diseases during the winter, can impact the saturation of emergency services, affecting response times and clinical outcomes [9,10]. A notable example is the COVID-19 pandemic, which highlighted a significant increase in mortality [11].

In addition to these temporal factors influencing mortality, it is crucial to consider the structural differences between emergency systems in different countries. In Spain, emergency services are predominantly centralized and managed by the public health system, whereas in the United States, they are more decentralized, with greater involvement from the private sector [12,13].

Therefore, a comprehensive consideration of all the factors described is essential to optimize the quality of prehospital care. Thus, the objectives of this study were to determine the associations between different time frames and mortality in patients treated by emergency systems and to consider the differences between the U.S. and Spanish health systems.

Despite the growing recognition of temporal factors in healthcare outcomes, there remains a significant knowledge gap regarding how these factors specifically affect prehospital emergency care across different health systems. Most existing research has focused on in-hospital settings, with limited comparative data examining temporal patterns in prehospital mortality between centralized public systems (like Spain) and more decentralized mixed public–private systems (like the U.S.). Understanding these patterns is crucial for developing evidence-based staffing models, resource allocation strategies, and quality improvement initiatives tailored to each system’s unique characteristics [1,5,12,13].

## 2. Materials and Methods

### 2.1. Study Design

This was a multicenter, EMS-based, observational study involving a prospective dataset, the Salud de Castilla y Leon dataset (Sacyl), and a retrospective dataset, the National Emergency Medical Services Information System (NEMSIS) [14].

For the Sacyl dataset, the study was approved by the local institutional research review board of the Public Health Service (reference: PI-049-19 and PI-GR-19-1258, on 5 March 2019), and the research protocol was registered with the WHO International Clinical Trials Registry Platform (ISRCTN48326533 and ISRCTN49321933). For NEMSIS, the institutional research granted a waiver/exemption owing to the use of deidentified data. We followed the guidelines of the Strengthening the Reporting of Observational Studies in Epidemiology (STROBE) Statement (Supplementary, p3) [15].

### 2.2. Study Settings

The Sacyl dataset was collected prospectively between 1 January 2018, and 31 December 2023, in four Spanish provinces (Burgos, Salamanca, Segovia and Valladolid). The EMS is operated by the public health system and is integrated by Advance Life Support (made up of 2 emergency medical technicians, an emergency registered nurse and a physician), Helicopter Emergency Medical Service (made up of an emergency registered nurse and a physician) and Basic Life Support (made up of 2 emergency medical technicians).

NEMSIS included retrospective data between 1 January 2018, and 31 December 2023, a United States of America-representative dataset of EMS activations populated by more than twelve thousand EMS agencies throughout the United States. The EMS included in the dataset is integrated with Advance Life Support (made up of 2 paramedics), Helicopter Emergency Medical Service (made up of an emergency registered nurse and/or a physician) and Basic Life Support (made up of 2 emergency medical technicians).

### 2.3. Population

All consecutive adult EMS activations (>18 years) evacuated with high priority to emergency departments were included in the analysis. Minors and all cases involving missing data were excluded.

### 2.4. Outcome

The primary outcome was short-term mortality assessed within different time windows for each cohort. For the Sacyl cohort, 2-day mortality (all-cause and in- and out-of-hospital) was reported, meaning all deaths occurring within 48 h of EMS activation, regardless of location, and for the NEMSIS cohort, short-term mortality was extrapolated from ED and hospital disposition (i.e., death recorded at the emergency department or upon hospital admission, without a fixed temporal window). While these outcome definitions are not strictly equivalent, both capture early mortality in the prehospital-to-hospital care continuum. Note that the high rate of missingness of outcome for NEMSIS is due to the structural lack of hospital-outcome linkage within the NEMSIS framework: (i) NEMSIS captures prehospital encounters, but definitive hospital outcomes are rarely integrated into the public research dataset. (ii) Many outcome-related fields are not mandatory for all reporting agencies, leading to systematic missingness.

### 2.5. Variable Selection

Note that Sacyl only collected two categories (rural or urban), and therefore, the wilderness and suburban categories from the NEMSIS dataset were recategorized into the two-category system: wilderness was mapped to rural, and suburban was mapped to urban. The time of attendance was separated into the following: shift (shift 1: 00:00:00–05:59:59, shift 2: 06:00:00–11:59:59, shift 3: 12:00:00–17:59:59, and shift 4: 18:00:00–23:59:59), day, month, and year.

### 2.6. Data Analysis

Descriptive results and the associations between the outcomes and the analyzed variables were assessed by the T-test, the Mann–Whitney U test or the chi–square test, when appropriate. Absolute values and percentages were used for categorical variables, and means and standard deviations were used for continuous variables. The effects of time (shift, day, month and year, each one was analyzed separately) on mortality were determined via a separate logistic regression for each cohort and time frame. Note that for time comparison, the first element of the timeframe (i.e., shift, Monday, January, and 2018) was used as a reference for comparisons.

All calculations and analyses were performed by using our own codes, R packages, and base functions in R, version 4.2.2 (http://www.R-project.org; accessed on 31 October 2022; the R Foundation for Statistical Computing, Vienna, Austria).

### 2.7. Ethics Approval

This study was approved by the Research Ethics Committee with Medicines of the Valladolid Health Area (Comité de Ética de la Investigación con Medicamentos, Área de Salud Valladolid), under approval codes PI-049-19 and PI-GR-19-1258, on 5 March 2019.

### 2.8. Clinical Trial Registration

This study was registered in the WHO International Clinical Trials Registry Platform (ISRCTN), under registration numbers ISRCTN48326533 (registered on 4 November 2019) and ISRCTN49321933 (registered on 25 February 2021).

This study is reported in accordance with the CONSORT 2025 statement.

## 3. Results

### Subsection

**Figure 1:**
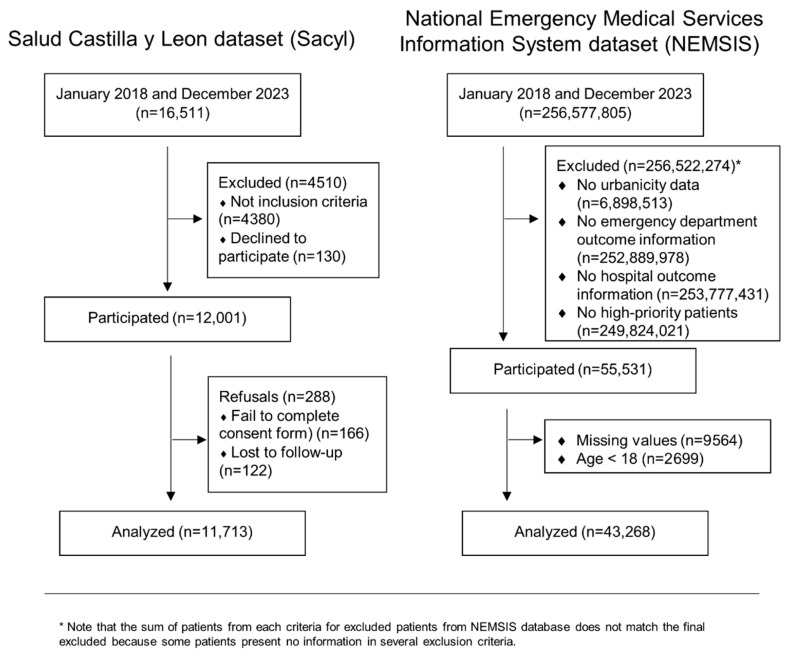
Flowchart.

**Figure 2:**
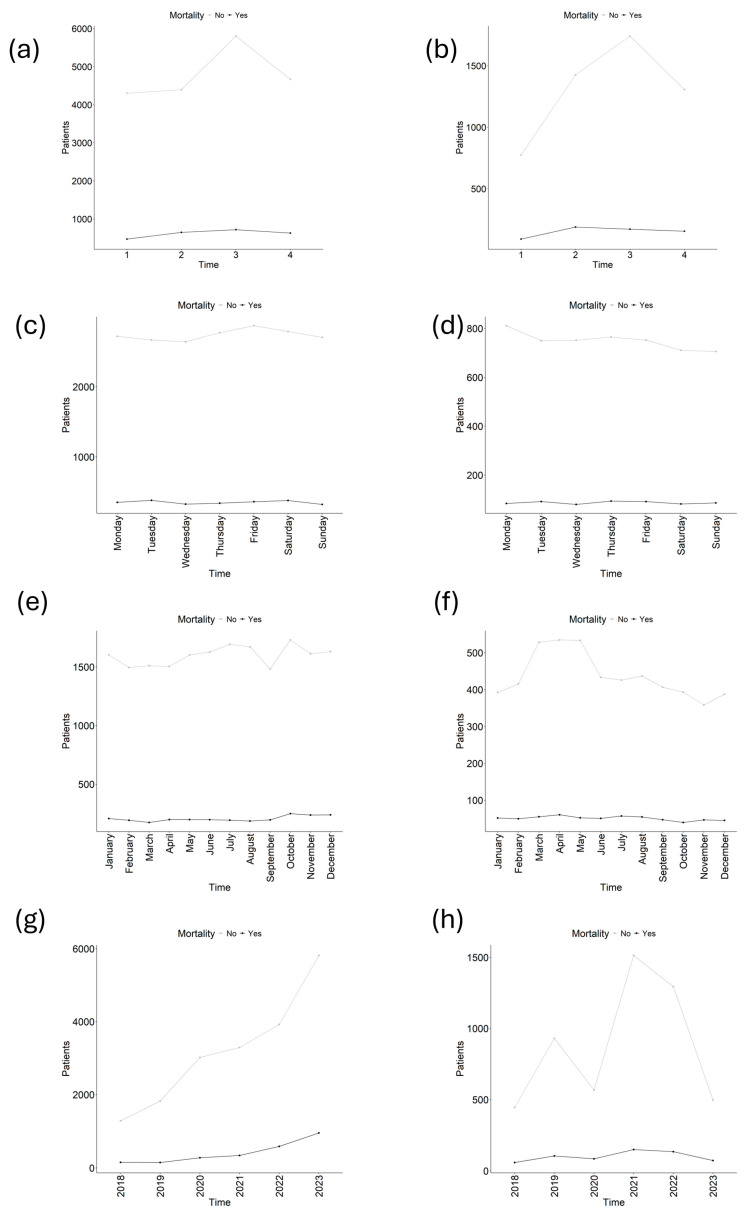
Number of patients according to time and mortality. (**a**) Shift for NEMSIS, (**b**) Shift for Sacyl, (**c**) day for NEMSIS, (**d**) day for Sacyl, (**e**) month for NEMSIS, (**f**) month for Sacyl, (**g**) year for NEMSIS, (**h**) year for Sacyl. Nonsurvivors = gray/lighter line, Survivors = black/darker line.

A total of 11,713 EMS activations were considered from the Sacyl dataset, and 43,268 were considered from the NEMSIS dataset (Figure 1). Compared with the Sacyl dataset, the NEMSIS dataset presented a greater absolute number of survivors and nonsurvivors on any shift day and month (Figure 2a, Figure 2b, Figure 2c and Figure 2d, respectively). A descriptive table according to mortality (Table 1) revealed that patients who died were older (67.3 (17.8) vs. 60.8 (19.7), *p* < 0.001), and 61.6% were men (*p* < 0.001). Mainly transported by advanced life support (ALS) (84.7%), *p* < 0.001, and living in urban areas (87.1%), *p* < 0.001. The main cause of complaint was disease (71.6%), affecting the cardiovascular system (45.2%), *p* < 0.001. Nonsurvivors presented higher rates of hospital and ICU admission (61.6% and 11.3%, respectively), both *p* < 0.001, and presented lower alert, support, transfer, and total times (all *p* < 0.001). A greater percentage of mortality occurred during the 06:00:00–11:59:59 and 18:00:00–23:59:59 shifts (both *p* < 0.001), on Monday (*p* = 0.025), and in 2022 (*p* = 0.029) and 2023 (*p* < 0.001). In contrast, mortality was lower in March (*p* = 0.034) and during years the 2019 (*p* < 0.001), 2020 (*p* = 0.004), and 2021 (*p* = 0.004). Note that raw percentages presented in Table 1 could be confusion and potentially contradictory to the logistic regression results and should be interpreted with care. Differences between cohorts are shown in Supplementary Table S1.

**Figure 3:**
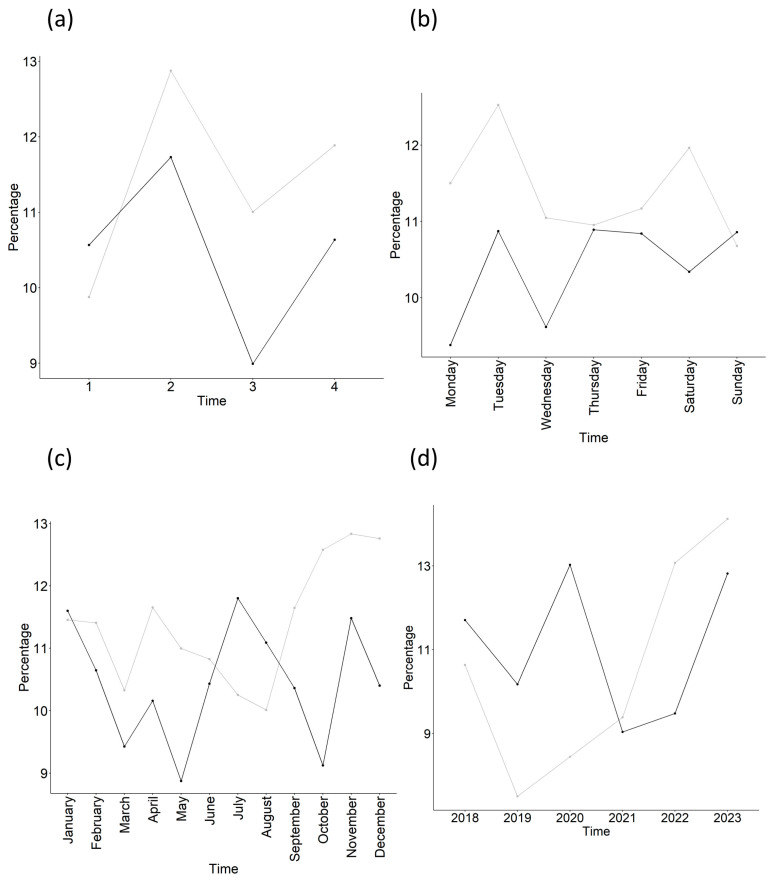
Percentages of nonsurvivors according to time and cohort. (**a**) Shift, (**b**) day, (**c**) month, (**d**) year. NEMSIS = gray/lighter line, Sacyl = black/darker line.

The percentage of mortality plots (Figure 3) revealed temporal patterns were similar for shift (Figure 3a), but not for day (Figure 3b), month (Figure 3c) or year (Figure 3d).

When focusing on each cohort, the NEMSIS (Supplementary Table S2) revealed that only shift and year presented statistically significant differences in mortality. This was also the case for Sacyl, in which the only significant differences in mortality were found for shift and year, but to a minor extent (Supplementary Table S3).

## 4. Discussion

This multicenter observational study, which was based on EMS data, used two large datasets: a prospective dataset from Sacyl and a retrospective dataset extracted from NEMSIS in the United States [14]. The primary objective was to evaluate the associations between different time frames (shift, day of the week, month, and year) and mortality in patients treated by emergency systems and compare the differences between the health systems of Spain and the U.S. The findings revealed that mortality varied significantly according to the time frame analyzed.

Overall, increased mortality was observed in men and older patients, which aligns with the literature on the vulnerability of these groups in severe medical emergencies [16]. There was also higher mortality in urban areas than in rural areas, despite the challenges of accessing quality care in the latter [17]. The study results indicate that mortality was significantly greater among patients requiring ALS, reflecting the severity of their underlying conditions. Cardiovascular diseases were identified as one of the main causes of mortality, suggesting greater vulnerability in emergency situations. This finding is consistent with previous research showing that these conditions not only increase the risk of mortality but also require intensive interventions, such as ALS, in critical cases [18].

With respect to the time frames, our results revealed significant variation in mortality depending on the time of day. Mortality is higher in the morning (06:00 to 12:00), decreases in the afternoon (12:00 to 18:00), and increases again in the evening (18:00 to 24:00). These findings are consistent with previous research suggesting that the demand for emergency services and the severity of cases fluctuate at different times of the day [19]. In line with our results, authors such as Nagarajan et al. [20] reported that most cases of acute myocardial infarction (AMI) occurred during the day, peaking at approximately 11 a.m. and decreasing at 4 a.m., highlighting the importance of morning hours in cardiovascular emergencies, which are a leading cause of mortality. This morning peak in AMI has been associated with physiological factors such as increased blood pressure and adrenaline levels, which can trigger critical events [21]. Furthermore, the circadian pattern of blood pressure, which increases upon waking and falls at night, contributes to greater cardiovascular instability during these hours of the day [22].

The higher demand for assistance during critical time slots, such as the morning, increases the workload. This can delay care, leading to a greater risk of mortality, especially in overburdened healthcare systems. High-activity periods put a strain on available resources, affecting both the speed and quality of care. Previous studies have indicated that increased workload in emergency services is associated with delays in care and a higher risk of mortality [4].

In summary, our results can be explained by the variation in the nature of emergencies and workload during each shift, which directly impacts the observed mortality. This underscores the importance of optimizing resource management and improving protocols during peak demand periods to reduce risks.

Additionally, significant differences were identified between the Sacyl and NEMSIS cohorts during the 12:00 to 18:00 shift, with higher mortality in the NEMSIS cohort. Several factors may contribute to this finding. First, the circadian pattern of cardiovascular events, particularly acute myocardial infarction, may extend later into the afternoon in the U.S. population compared to Spain, potentially due to differences in lifestyle patterns, meal timing, and work schedules. Second, the afternoon shift (12:00–18:00) often coincides with shift handover periods in many U.S. EMS systems, which could introduce care discontinuities or communication gaps that affect patient outcomes. Third, demographic differences and disparities in healthcare systems between the two countries may play a role, including differences in emergency department staffing patterns during afternoon hours. Previous studies have demonstrated that variations in healthcare system structure, resource availability, response time, and emergency care protocols can affect clinical outcomes and explain international disparities in mortality [23,24]. Specifically, comparative research between emergency systems has shown that countries with faster response times and access to specialized services have better survival rates for time-sensitive conditions [25]. Therefore, these differences between Sacyl and NEMSIS may be related to the efficiency of the emergency system and the characteristics of the populations served in each context.

With respect to the days of the week, the NEMSIS cohort had significantly greater mortality throughout the week (*p* = 0.011), which could indicate variability in emergency management or resource availability depending on the day. This suggests that mortality in NEMSIS may be influenced by factors such as workload, which could be related to the saturation of emergency services on certain days of the week in the U.S., a pattern previously documented in emergency service studies [26]. However, this trend observed in NEMSIS is not replicated when analyzed by other time frames, such as shifts, months, or years. This pattern suggests that factors affecting mortality throughout the week are not consistently present in other time periods, highlighting the importance of considering the weekly distribution of resources and operational management in the observed mortality. Importantly, our results did not support the existence of a classic “weekend effect” in this prehospital context. Using Monday as the reference category, neither Saturday nor Sunday was associated with an increased risk of mortality, suggesting that EMS activations during weekends were not linked to worse short-term outcomes in the studied populations. This finding should be interpreted considering several contextual factors. First, the study included exclusively high-priority patients, which may reduce care variability by focusing on time-sensitive emergencies managed under relatively standardized protocols. Second, outcome definitions differed between cohorts (fixed 48 h mortality in Sacyl versus emergency department or hospital disposition in NEMSIS), potentially attenuating subtle day-of-week differences. Finally, early mortality across the prehospital-to-hospital continuum may be more strongly driven by patient case mix and clinical severity than by operational changes related to the day of the week. Taken together, these results suggest that, within the limitations of this study, the weekend effect may not be generalizable to the specific setting of prehospital emergency care.

In terms of seasonality, no significant differences were found between the two cohorts, except in October, when mortality was higher in the NEMSIS cohort. This difference could be related to the earlier onset of respiratory infections in the U.S. than in Spain, as observed in studies on the seasonal patterns of these diseases [27]. Moreover, previous research has shown that the incidence of respiratory diseases can vary between regions due to climatic factors and differences in vaccination programs, which could also influence this disparity [28].

The year-by-year comparison revealed that, compared with 2018, 2019, 2020, 2022, and 2023 presented significant differences in mortality (*p* < 0.001). The increase in mortality in 2022 and 2023 can be attributed to the worsening of non-COVID-19 conditions and the exacerbation of untreated chronic conditions during the pandemic, a phenomenon supported by previous studies [29,30]. In particular, in 2020, mortality was higher in the Sacyl group (*p* = 0.011), which can be explained by the older population, which is thus more vulnerable to COVID-19 complications. However, in 2022, this trend reversed, with higher mortality in the NEMSIS group (*p* < 0.001). We hypothesized that this can be related to EMS operational strain, other unmeasured confounders, changes in coding practices, or, in the post-COVID-19 context, due to a worsening of non-COVID-19 conditions. However, this COVID-19-related hypothesis should be interpreted with caution, since no specific diagnoses (e.g., sepsis, late-stage cancer) were analyzed to prove it.

Finally, cohort analyses conducted with the NEMSIS and Sacyl indicate that the differences in mortality were exclusively associated with shift and year. This suggests that the time of day and year are the most relevant factors linked to mortality, which is supported by the consistent results from both datasets. This finding may indicate that workload and resource availability at specific times, as well as the evolution of public health in different years, significantly influence mortality outcomes. These patterns highlight the importance of considering these temporal factors when analyzing the effectiveness of emergency medical systems and their impact on mortality.

### Clinical and Operational Implications

The findings of this study have several important implications for clinical practice and emergency medical service operations. First, the identified peak mortality periods during the 06:00–12:00 and 18:00–24:00 shifts suggest that EMS systems should consider implementing dynamic staffing models that allocate more experienced clinicians and additional resources during these critical time windows. Healthcare administrators could use these temporal patterns to optimize crew composition, ensuring that advanced life support units with the most experienced emergency physicians and nurses are strategically deployed during high-risk periods. This targeted approach could potentially reduce response times and improve the quality of initial assessments and interventions during peak mortality hours, ultimately leading to better patient outcomes [2,4].

Second, the morning peak in mortality, which coincides with the circadian pattern of cardiovascular events, has direct implications for prehospital protocols. Emergency dispatch centers should consider implementing enhanced triage algorithms during morning hours (06:00–12:00) that prioritize rapid deployment of ALS resources for suspected cardiovascular emergencies. Additionally, prehospital providers should maintain heightened awareness of atypical presentations of acute myocardial infarction and stroke during these hours, as the physiological stress of morning awakening (including blood pressure surges and increased platelet aggregation) may contribute to more severe or rapidly evolving presentations [20,21,22]. This awareness could facilitate earlier recognition, more aggressive initial treatment, and expedited transport decisions during this critical period.

Third, the observed differences in afternoon mortality (12:00–18:00) between the U.S. and Spanish systems highlight the critical importance of shift handover protocols. The higher NEMSIS mortality during this period suggests that EMS systems, particularly those with multiple shift changeovers, should implement structured handoff procedures similar to those used in hospital settings. These protocols could include mandatory face-to-face briefings, standardized communication tools (such as SBAR—Situation, Background, Assessment, Recommendation), and brief overlapping shift periods to ensure continuity of care for ongoing cases. Furthermore, dispatch centers should track response times during shift transitions and implement compensatory measures (such as delayed shift changes or temporary resource redistribution) if performance metrics decline during these vulnerable periods.

Fourth, the seasonal variations identified, particularly the October mortality peak in the U.S., support the implementation of proactive seasonal preparedness strategies. EMS systems in regions prone to earlier respiratory infection seasons should consider pre-positioning additional resources, conducting targeted training refreshers on respiratory emergency management, and coordinating with public health authorities to align vaccination campaigns with EMS capacity planning. This anticipatory approach could include pre-winter inventory checks of respiratory support equipment, proactive maintenance of ventilators and oxygen delivery systems, and establishment of surge protocols that can be activated when seasonal respiratory illness indicators exceed predetermined thresholds [27,28].

The sustained increase in mortality observed during 2022 and 2023 likely reflects a post-pandemic phenomenon rather than an isolated temporal fluctuation. Beyond the direct impact of COVID-19, several studies have reported delayed presentations of acute conditions, poorer control of chronic diseases, and increased clinical severity at the time of emergency care in the post-pandemic period. These factors may have contributed to a higher short-term mortality among patients attended by emergency medical services during these years.

From a system-level perspective, emergency medical services may have experienced prolonged operational strain after the pandemic peak, including workforce shortages, increased workload, accumulated demand from deferred care, and ongoing reorganization of emergency care pathways. Such sustained pressure may negatively influence response times, clinical decision-making, and continuity of care, even after the acute phase of the pandemic had subsided. Alternatively, the observed increase in mortality may also indicate broader shifts in population health trends, such as population aging, higher prevalence of multimorbidity, and changes in health-seeking behaviors, with patients presenting later and in more severe conditions. Given the observational design of the study and the absence of diagnosis-specific or severity-adjusted data, these interpretations should be considered exploratory. Nevertheless, the consistent increase observed across cohorts highlights the importance of continued surveillance and further research to disentangle persistent post-pandemic effects from emerging population-level health changes [29,30].

Finally, these findings emphasize the importance of implementing comprehensive quality monitoring systems that track temporal patterns in clinical outcomes. EMS agencies should establish routine surveillance mechanisms to identify emerging temporal trends in mortality, allowing for rapid detection of system-level problems and timely implementation of corrective interventions. Such monitoring systems should include real-time dashboards displaying key performance indicators stratified by time of day, day of week, and season, with automated alerts when mortality rates exceed expected ranges for specific temporal periods. Regular quality improvement meetings should systematically review these temporal patterns, involve multidisciplinary teams (including dispatch, field providers, medical directors, and data analysts), and result in actionable interventions that can be evaluated for effectiveness. This data-driven approach to continuous improvement, grounded in temporal pattern recognition, represents a pathway toward more responsive, efficient, and ultimately more effective emergency medical systems that can adapt their operations to the varying demands and risks encountered across different time periods [1,4,5].

The main strengths of this work are the large number of patients and the diversity of health systems included. This enhances its generalizability since the conclusions could be interpreted considering both prehospital settings. Moreover, the analyzed years range from 2018 to 2023, including the COVID-19 pandemic period, which, at first glance, could be interpreted as bias. However, it should instead be seen as a reflection of how such a period affected health systems and how it continues to influence the following years, as seen in our results. Nonetheless, this study is not free of limitations. First, the variables selected for analysis were limited to those available in both datasets, which reduced the amount of information and prevented the inclusion of possible confounding factors. Second, in both cohorts, only high-priority patients were selected, leading to selection bias that should be considered when interpreting the results. Third, for the NEMSIS cohort, there was significant patient loss due to missing outcome information, which could introduce another selection bias. In large registries like NEMSIS, missingness may not be missing at random, potentially introducing selection bias (e.g., patients with rapid discharge or non-transport having less complete mortality data). However, given our massive sample size, we prioritize the use of observed, high-quality data. Fourth, the primary outcome definitions differed between cohorts: Sacyl used a fixed 2-day mortality window, while NEMSIS used hospital disposition status without a standardized temporal endpoint. This methodological inconsistency limits direct comparability and may introduce bias when interpreting differences between health systems. Fifth, the models did not include patient-level clinical severity indicators such as chief complaint severity, prehospital vital signs, or dispatch priority codes. The absence of these variables means that case-mix differences could confound the observed temporal patterns. Sixth, the outcome includes both in-hospital deaths and on-scene deaths; the former may be more influenced by in-hospital factors (e.g., emergency department crowding, staffing levels) than by EMS operational conditions, while the latter are largely determined by the patient’s pre-EMS condition rather than temporal EMS factors. Seventh, the Sacyl dataset was collected prospectively, whereas NEMSIS data were retrospective, which may affect data quality, completeness, and accuracy of recorded variables. Additionally, the recategorization of NEMSIS wilderness and suburban areas into rural and urban categories, respectively, may introduce misclassification bias, as suburban areas in the U.S. often have different EMS access and resources compared to truly urban settings, and wilderness areas face challenges distinct from standard rural environments.

Eighth, the study did not capture provider-level factors that could influence outcomes during different time periods, such as clinician fatigue, experience level variations across shifts, or differences in decision-making patterns that might occur as shifts progress. Night shift workers and those working extended hours may experience cognitive fatigue that affects clinical judgment, but our dataset did not include information on individual provider characteristics, shift schedules, or workload immediately preceding each emergency response. Similarly, the study could not account for the “July effect” in medical education systems, where the annual influx of new trainees might affect care quality in teaching hospitals and potentially influence outcomes for patients transported during certain months.

Ninth, we did not have access to data on specific prehospital interventions performed (such as advanced airway management, medication administration, or specific resuscitation procedures), which prevents us from determining whether temporal variations in mortality reflect differences in the aggressiveness of treatment, the appropriateness of interventions, or the technical success of procedures performed during different time periods. Understanding whether providers are more or less likely to attempt certain interventions during high-workload periods, and whether the success rates of these interventions vary by time of day, would provide valuable insights into the mechanisms underlying our observed temporal patterns.

Tenth, the analysis could not account for concurrent hospital-level factors that might contribute to temporal mortality patterns, such as variations in emergency department staffing, availability of specialty services (interventional cardiology, neurosurgery, intensive care beds), or differences in hospital protocols during different times of day or days of the week. Patients transported during certain time periods may be more likely to experience delays in definitive treatment due to hospital-level resource constraints, and these delays could contribute to the observed mortality patterns independently of prehospital factors. The interplay between prehospital and hospital factors in determining overall temporal patterns of mortality warrants further investigation through more comprehensive studies that capture both prehospital and in-hospital variables.

Eleventh, the study’s observational design precludes causal inference. While we identified associations between temporal factors and mortality, we cannot definitively establish that the time-of-day, seasonal, or annual patterns directly cause the observed differences in outcomes. Unmeasured confounding variables that correlate with both time periods and mortality risk could partially or fully explain the observed associations. For example, patients who experience emergencies during certain time periods may differ systematically in ways not captured by our available variables (such as socioeconomic status affecting when people seek care, behavioral factors like medication adherence patterns, or environmental exposures varying by season). Randomized controlled trials are not feasible for investigating temporal factors, so future research should employ more sophisticated causal inference methods, such as instrumental variable analysis or quasi-experimental designs, to strengthen conclusions about the causal nature of temporal effects on mortality.

Twelfth, the generalizability of our findings may be limited by the specific geographic regions included in each dataset. The Sacyl data come from four Spanish provinces (Burgos, Salamanca, Segovia, and Valladolid), which may not fully represent the diversity of Spanish emergency medical systems across all autonomous communities, particularly those in coastal or highly urbanized regions with different demographic profiles and healthcare infrastructures. Similarly, while NEMSIS represents a large and geographically diverse sample of U.S. EMS systems, the voluntary nature of NEMSIS participation and varying data quality across contributing agencies means that certain regions or types of EMS systems may be underrepresented. Rural, resource-limited, or smaller EMS agencies might be less likely to contribute comprehensive data to NEMSIS, potentially biasing the U.S. sample toward larger, more well-resourced systems with potentially different temporal mortality patterns than underrepresented systems. Thirtieth, the Global/General complaint category is the second most common. This category overrepresentation should be interpreted from a prehospital perspective, in which the diagnostic tools, techniques, etc., available in the hospital cannot be used. Therefore, these “global” categories are commonly used.

Finally, the study period spanning 2018–2023 encompasses extraordinary circumstances (the COVID-19 pandemic) that may limit the applicability of our findings to more stable epidemiological periods. The pandemic profoundly altered both the nature of emergency medical demand (with shifts in the types and severity of conditions presenting to EMS) and the operational context of emergency care (including modified protocols, resource constraints, and provider stress). While we attempted to account for annual variations, the complex and evolving nature of pandemic impacts may not be fully captured by simple year-to-year comparisons. The observed patterns during 2020–2023 may reflect transient phenomena specific to the pandemic era rather than enduring characteristics of temporal variations in prehospital mortality. Future studies conducted during post-pandemic periods will be essential to determine which of our observed temporal patterns represent persistent features of emergency medical care and which were unique to this extraordinary historical period. Despite these limitations, the study’s strengths—including large sample sizes, diverse health system representation, and comprehensive temporal coverage—provide valuable insights that can inform emergency medical service operations and identify important directions for future research.

## 5. Conclusions

In conclusion, this study demonstrated an association of temporal factors such as the time of day and the year with mortality in emergency medical systems. These results highlight the need to adjust emergency service strategies, taking these temporal and geographical factors into account, to optimize resources and improve clinical outcomes.

## Figures and Tables

**Table 1:** Patient characteristics according to mortality. Note that statistical report of massive data should be better interpreted considering odds ratios rather than percentages or *p*-values.

	Survivors	Nonsurvivors	Odds Ratio [95%CI]	*p* Value
	N = 48,828	N = 6153		
age	60.8 (19.7)	67.3 (17.8)	1.02 [1.02;1.02]	<0.001
Sex:				
Female	20,736 (42.5%)	2363 (38.4%)	Ref.	Ref.
Male	28,092 (57.5%)	3790 (61.6%)	1.18 [1.12;1.25]	<0.001
Level of Care of This Unit:				
No-Advanced life support	8362 (17.1%)	939 (15.3%)	Ref.	Ref.
Yes-Advanced life support	40,466 (82.9%)	5214 (84.7%)	1.15 [1.07;1.24]	<0.001
Zone:				
Rural	8588 (17.6%)	795 (12.9%)	Ref.	Ref.
Urban	40,240 (82.4%)	5358 (87.1%)	1.44 [1.33;1.56]	<0.001
Complaint_Reported_by_Dispatch:				
causal_accident	6832 (14.0%)	608 (9.88%)	Ref.	Ref.
Disease	27,603 (56.5%)	4407 (71.6%)	1.79 [1.64;1.96]	<0.001
Occupational accident	186 (0.38%)	14 (0.23%)	0.85 [0.47;1.43]	0.568
Others	5301 (10.9%)	555 (9.02%)	1.18 [1.04;1.33]	0.008
Social welfare calls	6179 (12.7%)	302 (4.91%)	0.55 [0.48;0.63]	<0.001
Traffic	2727 (5.58%)	267 (4.34%)	1.10 [0.95;1.28]	0.215
Chief Complaint Organ System:				
Cardiovascular	11,529 (23.6%)	2779 (45.2%)	Ref.	Ref.
Endocrine_Metabolic	928 (1.90%)	54 (0.88%)	0.24 [0.18;0.32]	<0.001
Gastrointestinal	1432 (2.93%)	95 (1.54%)	0.28 [0.22;0.34]	<0.001
Genitourinary	369 (0.76%)	4 (0.07%)	0.05 [0.01;0.11]	<0.001
Global_General	14,667 (30.0%)	1593 (25.9%)	0.45 [0.42;0.48]	<0.001
Lymphatic/Immune	807 (1.65%)	181 (2.94%)	0.93 [0.79;1.10]	0.398
Musculoskeletal/Skin/Trauma	5081 (10.4%)	301 (4.89%)	0.25 [0.22;0.28]	<0.001
Neurologic	9385 (19.2%)	723 (11.8%)	0.32 [0.29;0.35]	<0.001
Pulmonary	4630 (9.48%)	423 (6.87%)	0.38 [0.34;0.42]	<0.001
Hospital-admission:				
No	25,053 (51.3%)	2365 (38.4%)	Ref.	Ref.
Yes	23,775 (48.7%)	3788 (61.6%)	1.69 [1.60;1.78]	<0.001
ICU-admission:				
No	44,246 (90.6%)	5455 (88.7%)	Ref.	Ref.
Yes	4582 (9.38%)	698 (11.3%)	1.24 [1.13;1.34]	<0.001
Alert time	13.7 (25.9)	10.2 (11.7)	0.98 [0.98;0.98]	<0.001
Support time	23.7 (17.6)	26.1 (16.1)	1.01 [1.01;1.01]	<0.001
Transfer time	17.6 (19.9)	13.2 (10.8)	0.98 [0.98;0.98]	<0.001
Total time	55.0 (45.3)	49.5 (27.1)	1.00 [1.00;1.00]	<0.001
Shift:				
00:00:00–05:59:59	10,154 (20.8%)	1126 (18.3%)	Ref.	Ref.
06:00:00–11:59:59	11,641 (23.8%)	1678 (27.3%)	1.30 [1.20;1.41]	<0.001
12:00:00–17:59:59	15,087 (30.9%)	1779 (28.9%)	1.06 [0.98;1.15]	0.126
18:00:00–23:59:59	11,946 (24.5%)	1570 (25.5%)	1.19 [1.09;1.29]	<0.001
Day:				
Monday	7063 (14.5%)	875 (14.2%)	Ref.	Ref.
Tuesday	6835 (14.0%)	947 (15.4%)	1.12 [1.01;1.23]	0.025
Wednesday	6786 (13.9%)	816 (13.3%)	0.97 [0.88;1.07]	0.563
Thursday	7074 (14.5%)	869 (14.1%)	0.99 [0.90;1.10]	0.868
Friday	7247 (14.8%)	905 (14.7%)	1.01 [0.91;1.11]	0.874
Saturday	6999 (14.3%)	922 (15.0%)	1.06 [0.96;1.17]	0.220
Sunday	6824 (14.0%)	819 (13.3%)	0.97 [0.88;1.07]	0.538
Month:				
January	3993 (8.18%)	518 (8.42%)	Ref.	Ref.
February	3821 (7.83%)	484 (7.87%)	0.98 [0.86;1.11]	0.723
March	4078 (8.35%)	458 (7.44%)	0.87 [0.76;0.99]	0.034
April	4080 (8.36%)	518 (8.42%)	0.98 [0.86;1.11]	0.744
May	4273 (8.75%)	500 (8.13%)	0.90 [0.79;1.03]	0.121
June	4121 (8.44%)	496 (8.06%)	0.93 [0.81;1.06]	0.261
July	4239 (8.68%)	501 (8.14%)	0.91 [0.80;1.04]	0.161
August	4217 (8.64%)	481 (7.82%)	0.88 [0.77;1.00]	0.055
September	3779 (7.74%)	485 (7.88%)	0.99 [0.87;1.13]	0.873
October	4248 (8.70%)	577 (9.38%)	1.05 [0.92;1.19]	0.476
November	3943 (8.08%)	568 (9.23%)	1.11 [0.98;1.26]	0.106
December	4036 (8.27%)	567 (9.22%)	1.08 [0.95;1.23]	0.219
Year:				
2018	3470 (7.11%)	425 (6.91%)	Ref.	Ref.
2019	5512 (11.3%)	507 (8.24%)	0.75 [0.66;0.86]	<0.001
2020	7172 (14.7%)	727 (11.8%)	0.83 [0.73;0.94]	0.004
2021	9611 (19.7%)	983 (16.0%)	0.83 [0.74;0.94]	0.004
2022	10,434 (21.4%)	1451 (23.6%)	1.14 [1.01;1.27]	0.029
2023	12,629 (25.9%)	2060 (33.5%)	1.33 [1.19;1.49]	<0.001
cohort:				
NEMSIS	38,332 (78.5%)	4936 (80.2%)	Ref.	Ref.
Sacyl	10,496 (21.5%)	1217 (19.8%)	0.90 [0.84;0.96]	0.002

## Data Availability

The data presented in this study are available on request from the corresponding authors. Because the data are not publicly available due to privacy or ethical restrictions.

## Supplementary Materials

The following supporting information can be downloaded at: https://www.mdpi.com/article/10.3390/medsci14010056/s1, p3: STROBE1 Statement—checklist of items that should be included in reports of observational studies; Table S1: Patients’ characteristics according to cohort; Table S2: Association between mortality and time for NEMSIS; Table S3: Association between mortality and time for Sacyl.

## Abbreviations

The following abbreviations are used in this manuscript:

ALS	Advanced Life Support
BLS	Basic Life Support
ED	Emergency Department
EMS	Emergency Medical Services
