# Supplementary material for: Association of Shift, Day, Month and Year with Mortality: Observational Study of Spanish and USA Emergency Care Cohorts
Source: Med Sci (Basel). 2026 Jan 22;14(1):56. doi: 10.3390/medsci14010056 (PMC12922032; doi:10.3390/medsci14010056)
Supplement: Supplementary file 1 [file medsci-14-00056-s001.zip › medsci-4091669-supplementary.pdf]

Supplementary data

Table of contents

Supplementary methods……………………………………………………………………………3
Strengthening the Reporting of Observational Studies in Epidemiology (STROBE) Statement……………………..…………………………………3
Data collection, Missing values, sample size calculation, software…...………..5
Supplementary Results…..……………………………………………………………..6
- Supplementary table S1. Patients' characteristics according to cohort………..6
- Supplementary table S2. Association between mortality and time for NEMSIS……………………………………………………………………...….9
- Supplementary table S3. Association between mortality and time for Sacyl...10
References...11

Supplementary Methods

STROBE[1] Statement—checklist of items that should be included in reports of observational studies

	Item No	Recommendation	Page No
**Title and abstract**	1	(*a*) Indicate the study's design with a commonly used term in the title or the abstract	1
		(*b*) Provide in the abstract an informative and balanced summary of what was done and what was found	3
**Introduction**			
Background/rationale	2	Explain the scientific background and rationale for the investigation being reported	4
Objectives	3	State specific objectives, including any prespecified hypotheses	4
**Methods**			
Study design	4	Present key elements of study design early in the paper	6
Setting	5	Describe the setting, locations, and relevant dates, including periods of recruitment, exposure, follow-up, and data collection	6
Participants	6	(*a*) *Cohort study*—Give the eligibility criteria, and the sources and methods of selection of participants. Describe methods of follow-up<br>*Case-control study*—Give the eligibility criteria, and the sources and methods of case ascertainment and control selection. Give the rationale for the choice of cases and controls<br>*Cross-sectional study*—Give the eligibility criteria, and the sources and methods of selection of participants	6
		(*b*) *Cohort study*—For matched studies, give matching criteria and number of exposed and unexposed<br>*Case-control study*—For matched studies, give matching criteria and the number of controls per case	6
Variables	7	Clearly define all outcomes, exposures, predictors, potential confounders, and effect modifiers. Give diagnostic criteria, if applicable	6
Data sources/ measurement	8*	For each variable of interest, give sources of data and details of methods of assessment (measurement). Describe comparability of assessment methods if there is more than one group	*6*
Bias	9	Describe any efforts to address potential sources of bias	7
Study size	10	Explain how the study size was arrived at	7
Quantitative variables	11	Explain how quantitative variables were handled in the analyses. If applicable, describe which groupings were chosen and why	7
Statistical methods	12	(*a*) Describe all statistical methods, including those used to control for confounding	7
		(*b*) Describe any methods used to examine subgroups and interactions	7
		(*c*) Explain how missing data were addressed	7
		(*d*) *Cohort study*—If applicable, explain how loss to follow-up was addressed<br>*Case-control study*—If applicable, explain how matching of cases and controls was addressed<br>*Cross-sectional study*—If applicable, describe analytical methods taking account of sampling strategy	7
		(*e*) Describe any sensitivity analyses	7

Continued on next page

Results

Participants	13*	(a) Report numbers of individuals at each stage of study—eg numbers potentially eligible, examined for eligibility, confirmed eligible, included in the study, completing follow-up, and analysed	9
		(b) Give reasons for non-participation at each stage	9
		(c) Consider use of a flow diagram	9
Descriptive data	14*	(a) Give characteristics of study participants (eg demographic, clinical, social) and information on exposures and potential confounders	9
		(b) Indicate number of participants with missing data for each variable of interest	9
		(c) *Cohort study*—Summarise follow-up time (eg, average and total amount)	9
Outcome data	15*	*Cohort study*—Report numbers of outcome events or summary measures over time	*9*
		Case-control study—Report numbers in each exposure category, or summary measures of exposure	*9*
		Cross-sectional study—Report numbers of outcome events or summary measures	*9*
Main results	16	(*a*) Give unadjusted estimates and, if applicable, confounder-adjusted estimates and their precision (eg, 95% confidence interval). Make clear which confounders were adjusted for and why they were included	9
		(*b*) Report category boundaries when continuous variables were categorized	9
		(*c*) If relevant, consider translating estimates of relative risk into absolute risk for a meaningful time period	9
Other analyses	17	Report other analyses done—eg analyses of subgroups and interactions, and sensitivity analyses	9
**Discussion**			
Key results	18	Summarise key results with reference to study objectives	11-12
Limitations	19	Discuss limitations of the study, taking into account sources of potential bias or imprecision. Discuss both direction and magnitude of any potential bias	12-13
Interpretation	20	Give a cautious overall interpretation of results considering objectives, limitations, multiplicity of analyses, results from similar studies, and other relevant evidence	11,12,13
Generalisability	21	Discuss the generalisability (external validity) of the study results	11,12,13
**Other information**			
Funding	22	Give the source of funding and the role of the funders for the present study and, if applicable, for the original study on which the present article is based	2

*Give information separately for cases and controls in case-control studies and, if applicable, for exposed and unexposed groups in cohort and cross-sectional studies.

Note: An Explanation and Elaboration article discusses each checklist item and gives methodological background and published examples of transparent reporting. The STROBE checklist is best used in conjunction with this article (freely available on the Web sites of PLoS Medicine at http://www.plosmedicine.org/, Annals of Internal Medicine at http://www.annals.org/, and Epidemiology at http://www.epidem.com/). Information on the STROBE Initiative is available at www.strobe-statement.org.

Data collection and missing values
Sacyl data was collected and registered in a database generated with the IBM SPSS Statistics for Apple version 20.0 software. (IBM Corp, Armonk USA). The caseload entry system was test-run to delete unclear or ambiguous items and to verify the adequacy of the data gathering system. NEMSIS Data was collected from NEMSIS (https://nemsis.org/). The data present missing values but as occurred in the Sacyl database, missing values were completely at random.

Sample Size
Because this study utilized a comprehensive national registry and a specific database, the sample size was predetermined by the total number of eligible patients during the study period. A formal power analysis was not performed a priori as the study aimed to maximize the use of all available data to provide the highest possible precision for population-level estimates.

Software
All calculations and analyses were performed by using our own codes, R packages and base functions in R, version 4.2.2 (http://www.R-project.org; the R Foundation for Statistical Computing, Vienna, Austria).

Supplementary table S1. Patients' characteristics according to cohort

	NEMSIS N=43268	Sacyl N=11713	Odds ratio [95%CI]	pvalue
age	60.6 (19.6)	64.9 (19.2)	1.01 [1.01;1.01]	<0.001
Sex:				
Female	18223 (42.1%)	4876 (41.6%)	Ref.	Ref.
Male	25045 (57.9%)	6837 (58.4%)	1.02 [0.98;1.06]	0.343
Level of Care of This Unit:				
No-Advanced life support	4930 (11.4%)	4371 (37.3%)	Ref.	Ref.
Yes-Advanced life support	38338 (88.6%)	7342 (62.7%)	0.22 [0.21;0.23]	<0.001
Zone:				
Rural	7069 (16.3%)	2314 (19.8%)	Ref.	Ref.
Urban	36199 (83.7%)	9399 (80.2%)	0.79 [0.75;0.84]	<0.001
Complaint_Reported_by_Dispatch:				
causal_accident	6898 (15.9%)	542 (4.63%)	Ref.	Ref.
Disease	22536 (52.1%)	9474 (80.9%)	5.35 [4.89;5.86]	<0.001
Occupational accident	12 (0.03%)	188 (1.61%)	197 [114;375]	<0.001
Others	5769 (13.3%)	87 (0.74%)	0.19 [0.15;0.24]	<0.001
Social welfare calls	5684 (13.1%)	797 (6.80%)	1.78 [1.59;2.00]	<0.001
Traffic	2369 (5.48%)	625 (5.34%)	3.36 [2.97;3.80]	<0.001
Chief Complaint Organ System:				
Cardiovascular	9668 (22.3%)	4640 (39.6%)	Ref.	Ref.
Endocrine_Metabolic	771 (1.78%)	211 (1.80%)	0.57 [0.49;0.67]	<0.001
Gastrointestinal	1006 (2.33%)	521 (4.45%)	1.08 [0.96;1.21]	0.181
Genitourinary	341 (0.79%)	32 (0.27%)	0.20 [0.13;0.28]	<0.001
Global_General	15325 (35.4%)	935 (7.98%)	0.13 [0.12;0.14]	<0.001
Lymphatic/Immune	229 (0.53%)	759 (6.48%)	6.90 [5.94;8.05]	<0.001
Musculoskeletal/Skin/Trauma	3869 (8.94%)	1513 (12.9%)	0.81 [0.76;0.87]	<0.001
Neurologic	7870 (18.2%)	2238 (19.1%)	0.59 [0.56;0.63]	<0.001
Pulmonary	4189 (9.68%)	864 (7.38%)	0.43 [0.40;0.47]	<0.001
Hospital-admission:				
No	22060 (51.0%)	5358 (45.7%)	Ref.	Ref.
Yes	21208 (49.0%)	6355 (54.3%)	1.23 [1.18;1.29]	<0.001

	NEMSIS N=43268	Sacyl N=11713	Odds ratio [95%CI]	pvalue
ICU-admission:				
No	40654 (94.0%)	9047 (77.2%)	Ref.	Ref.
Yes	2614 (6.04%)	2666 (22.8%)	4.58 [4.32;4.86]	<0.001
Mortality:				
No	38332 (88.6%)	10496 (89.6%)	Ref.	Ref.
Yes	4936 (11.4%)	1217 (10.4%)	0.90 [0.84;0.96]	0.002
Alert time	13.6 (27.7)	12.2 (7.29)	1.00 [1.00;1.00]	<0.001
Support time	22.5 (18.7)	29.3 (10.3)	1.02 [1.02;1.02]	<0.001
Transfer time	18.4 (20.9)	12.5 (8.84)	0.97 [0.97;0.97]	<0.001
Total time	54.4 (48.4)	54.0 (17.1)	1.00 [1.00;1.00]	0.374
shift:				
00:00:00-05:59:59	9548 (22.1%)	1732 (14.8%)	Ref.	Ref.
06:00:00-11:59:59	10088 (23.3%)	3231 (27.6%)	1.77 [1.66;1.88]	<0.001
12:00:00-17:59:59	13040 (30.1%)	3826 (32.7%)	1.62 [1.52;1.72]	<0.001
18:00:00-23:59:59	10592 (24.5%)	2924 (25.0%)	1.52 [1.43;1.63]	<0.001
weekday:				
Monday	6147 (14.2%)	1791 (15.3%)	Ref.	Ref.
Tuesday	6099 (14.1%)	1683 (14.4%)	0.95 [0.88;1.02]	0.158
Wednesday	5938 (13.7%)	1664 (14.2%)	0.96 [0.89;1.04]	0.313
Thursday	6226 (14.4%)	1717 (14.7%)	0.95 [0.88;1.02]	0.151
Friday	6464 (14.9%)	1688 (14.4%)	0.90 [0.83;0.97]	0.004
Saturday	6335 (14.6%)	1586 (13.5%)	0.86 [0.80;0.93]	<0.001
Sunday	6059 (14.0%)	1584 (13.5%)	0.90 [0.83;0.97]	0.005
month:				
January	3623 (8.37%)	888 (7.58%)	Ref.	Ref.
February	3375 (7.80%)	930 (7.94%)	1.12 [1.01;1.25]	0.026
March	3369 (7.79%)	1167 (9.96%)	1.41 [1.28;1.56]	<0.001
April	3407 (7.87%)	1191 (10.2%)	1.43 [1.29;1.57]	<0.001
May	3601 (8.32%)	1172 (10.0%)	1.33 [1.20;1.47]	<0.001
June	3649 (8.43%)	968 (8.26%)	1.08 [0.98;1.20]	0.129
July	3774 (8.72%)	966 (8.25%)	1.04 [0.94;1.16]	0.404
August	3715 (8.59%)	983 (8.39%)	1.08 [0.98;1.20]	0.140

	NEMSIS N=43268	Sacyl N=11713	Odds ratio [95%CI]	pvalue
September	3357 (7.76%)	907 (7.74%)	1.10 [0.99;1.22]	0.066
October	3959 (9.15%)	866 (7.39%)	0.89 [0.80;0.99]	0.032
November	3701 (8.55%)	810 (6.92%)	0.89 [0.80;0.99]	0.036
December	3738 (8.64%)	865 (7.38%)	0.94 [0.85;1.05]	0.280
year:				
2018	2887 (6.67%)	1008 (8.61%)	Ref.	Ref.
2019	3946 (9.12%)	2073 (17.7%)	1.50 [1.38;1.65]	<0.001
2020	6594 (15.2%)	1305 (11.1%)	0.57 [0.52;0.62]	<0.001
2021	7264 (16.8%)	3330 (28.4%)	1.31 [1.21;1.43]	<0.001
2022	9027 (20.9%)	2858 (24.4%)	0.91 [0.83;0.99]	0.021
2023	13550 (31.3%)	1139 (9.72%)	0.24 [0.22;0.26]	<0.001

Supplementary table S2. Association between mortality and time for NEMSIS

Shift

	Estimate	Std. Error	z value	Pvalue
Shift2	0.29913	0.04539	6.590	<0.001
Shift3	0.12076	0.04427	2.728	0.006
Shift4	0.20779	0.04558	4.559	<0.001

Weekday

	Estimate	Std. Error	z value	Pvalue
TimeTuesday	0.09703	0.05563	1.744	0.0811
TimeWednesday	-0.04540	0.05755	-0.789	0.4302
TimeThursday	-0.05494	0.05696	-0.964	0.3348
TimeFriday	-0.03303	0.05619	-0.588	0.5566
TimeSaturday	0.04478	0.05565	0.805	0.4210
TimeSunday	-0.08352	0.05769	-1.448	0.1477

Month

	Estimate	Std. Error	z value	Pvalue
TimeFebruary	-0.004661	0.075187	-0.062	0.9506
TimeMarch	-0.116016	0.076977	-1.507	0.1318
TimeApril	0.019365	0.074648	0.259	0.7953
TimeMay	-0.045929	0.074555	-0.616	0.5379
TimeJune	-0.063630	0.074566	-0.853	0.3935
TimeJuly	-0.124151	0.074834	-1.659	0.0971
TimeAugust	-0.150606	0.075552	-1.993	0.0462
TimeSeptember	0.018862	0.074939	0.252	0.8013
TimeOctober	0.106412	0.070839	1.502	0.1331
TimeNovember	0.129441	0.071669	1.806	0.0709
TimeDecember	0.122852	0.071585	1.716	0.0861

Year

	Estimate	Std. Error	z value	Pvalue
Time2019	-0.38343	0.08542	-4.489	<0.001
Time2020	-0.25440	0.07487	-3.398	<0.001
Time2021	-0.13837	0.07255	-1.907	0.05648
Time2022	0.23408	0.06797	3.444	<0.001
Time2023	0.32379	0.06522	4.965	<0.001

Supplementary table S3. Association between mortality and time for Sacyl

Shift

	**Estimate**	**Std. Error**	**z value**	**Pvalue**
Shift2	0.117639	0.095390	1.233	0.2175
Shift3	-0.178842	0.096458	-1.854	0.0637
Shift4	0.007417	0.098530	0.075	0.9400

Weekday

	**Estimate**	**Std. Error**	**z value**	**Pvalue**
TimeTuesday	0.16433	0.11268	1.458	0.145
TimeWednesday	0.02736	0.11610	0.236	0.814
TimeThursday	0.16615	0.11210	1.482	0.138
TimeFriday	0.16101	0.11267	1.429	0.153
TimeSaturday	0.10811	0.11561	0.935	0.350
TimeSunday	0.16280	0.11440	1.423	0.155

Month

	**Estimate**	**Std. Error**	**z value**	**Pvalue**
TimeFebruary	-0.09656	0.14928	-0.647	0.5178
TimeMarch	-0.23175	0.14497	-1.599	0.1099
TimeApril	-0.14867	0.14206	-1.047	0.2953
TimeMay	-0.29820	0.14673	-2.032	0.0421
TimeJune	-0.11896	0.14844	-0.801	0.4229
TimeJuly	0.01957	0.14466	0.135	0.8924
TimeAugust	-0.05078	0.14595	-0.348	0.7279
TimeSeptember	-0.12648	0.15116	-0.837	0.4027
TimeOctober	-0.26783	0.15782	-1.697	0.0897
TimeNovember	-0.01152	0.15208	-0.076	0.9396
TimeDecember	-0.12210	0.15291	-0.798	0.4246

Year

	Estimate	Std. Error	z value	**Pvalue**
Time2019	-0.15701	0.12196	-1.287	0.1980
Time2020	0.12195	0.12791	0.953	0.3404
Time2021	-0.28834	0.11511	-2.505	0.0122
Time2022	-0.23560	0.11694	-2.015	0.0439
Time2023	0.10341	0.13212	0.783	0.4338

References

1. Vandenbroucke JP. von Elm E. Altman DG. Gøtzsche PC. Mulrow CD. Pocock SJ. Poole C. Schlesselman JJ. Egger M; STROBE Initiative. Strengthening the Reporting of Observational Studies in Epidemiology (STROBE): explanation and elaboration. PLoS Med. 2007 Oct 16;4(10):e297.
